# Photodynamic Therapy for Keratinocytic Precancerous Lesions and Non-Melanoma Skin Cancer: A Narrative Review

**DOI:** 10.3390/ijms27146396

**Published:** 2026-07-18

**Authors:** Francesco Russano, Luigi Dall’Olmo, Davide Brugnolo, Francesco Callegarin, Paolo Del Fiore, Rocco Caminiti, Marco Rastrelli, Simone Mocellin

**Affiliations:** 1 Soft-Tissue, Peritoneum and Melanoma Surgical Oncology Unit, Veneto Institute of Oncology IOV—IRCCS, 35128 Padua, Italy; francesco.russano@iov.veneto.it (F.R.); luigi.dallolmo@unipd.it (L.D.); paolo.delfiore@iov.veneto.it (P.D.F.); marco.rastrelli@unipd.it (M.R.); simone.mocellin@unipd.it (S.M.); 2 Department of Surgery, Oncology and Gastroenterology (DISCOG), University of Padua, 35020 Padua, Italy; davide.brugnolo@iov.veneto.it; 3 Clinical Research Unit, Veneto Institute of Oncology IOV—IRCCS, 35128 Padua, Italy; 4 Casa di Cura Caminiti, Villa San Giovanni, 89018 Reggio Calabria, Italy; rocco.caminiti@icloud.it

**Keywords:** photodynamic therapy, actinic keratosis, field cancerization, basal cell carcinoma, Bowen disease, squamous cell carcinoma, non-melanoma skin cancer, photosensitizing agents

## Abstract

Photodynamic therapy (PDT) is a cornerstone non-invasive modality for keratinocytic precancers and non-melanoma skin cancer (NMSC), leveraging selective photosensitizer accumulation, light activation, and reactive oxygen species (ROS) generation. This narrative review synthesized literature from major databases (2010–2025) to comprehensively evaluate PDT’s molecular mechanisms, innovative optimization protocols, and clinical efficacy across actinic keratosis (AK), field cancerization, Bowen’s disease (BD), basal cell carcinoma (BCC), and invasive squamous cell carcinoma (cSCC). The evidence highlights frontline clinical maturity and excellent cosmetic outcomes for superficial lesions (AK, field cancerization, superficial BCC, and BD), with daylight PDT offering a virtually painless alternative for widespread dysplasia. However, therapeutic reliability decreases in thick nodular, pigmented, or high-risk lesions due to optical barriers and tissue hypoxia. To overcome these limitations, advanced physical and chemical enhancements—such as ablative fractional lasers, iron chelators, epigenetically enhanced PDT (ePDT), and targeted nanocarriers—are actively reshaping drug delivery and cellular susceptibility. Furthermore, cyclic PDT serves as an indispensable tissue-sparing intervention for organ transplant recipients and Gorlin syndrome patients. In conclusion, while PDT is highly effective for superficial neoplasias, precise histopathological stratification and the integration of nanomedicine are critical to overcoming current biological barriers in aggressive dermatological malignancies.

## 1. Introduction

Non-melanoma skin cancer (NMSC) represents the most common group of malignancies in Caucasian populations, predominantly comprising basal cell carcinoma (BCC) and cutaneous squamous cell carcinoma (cSCC) [1,2]. Driven by aging populations and cumulative exposure to ultraviolet (UV) radiation, the global incidence of NMSCs is steadily increasing, imposing a substantial burden on healthcare systems [1,3,4]. Actinic keratoses (AKs) are highly prevalent precancerous lesions arising on chronically sun-damaged skin, and they carry a significant, albeit variable, risk of progression into invasive cSCC [5,6]. Furthermore, chronic UV exposure often induces “field cancerization,” a process where clinically normal-appearing skin surrounding visible lesions harbors subclinical, multifocal genetic and cellular alterations that predispose the area to the continuous development of new primary tumors [5,6]. While surgical excision remains the traditional gold standard for the treatment of invasive NMSCs due to its high clearance rates and histological margin control [2,7], it can cause significant patient morbidity, scarring, and disfigurement. Surgery is also frequently impractical for patients presenting with multiple lesions, widespread field cancerization, or tumors located in cosmetically sensitive areas [7,8]. Consequently, a variety of non-surgical field- and lesion-directed therapies have been developed, including cryotherapy and topical chemotherapeutics such as 5-fluorouracil, imiquimod, and diclofenac [5,9]. Among these, Photodynamic Therapy (PDT) has firmly established itself as a frontline, non-invasive treatment modality, highly valued for its selective destruction of diseased tissue, repeatability, and excellent cosmetic outcomes [10,11,12]. At its core, PDT relies on the synergistic interaction of three essential components: a non-toxic photosensitizer (PS) or prodrug, light of a specific wavelength, and molecular oxygen [13,14]. In dermatological oncology, the most widely utilized agents are the topically applied prodrugs 5-aminolevulinic acid (5-ALA) and its more lipophilic ester, methyl aminolevulinate (MAL) [11,15]. Due to the altered metabolism of neoplastic cells and an enzymatic bottleneck at ferrochelatase, these precursors selectively accumulate within the mitochondria of target cells and are converted into protoporphyrin IX (PpIX), a highly fluorescent, endogenous photosensitizer [13,16,17]. Upon illumination with a targeted light source (most commonly red or blue light), PpIX transitions to an excited triplet state and initiates two primary photochemical pathways: Type I reactions, which involve electron transfer to generate free radicals, and the predominant Type II reactions, which directly transfer energy to molecular oxygen to produce highly reactive singlet oxygen [8,18]. These reactive oxygen species (ROS) induce lethal oxidative stress, leading to targeted cell death through apoptosis, necrosis, and autophagy. Concurrently, PDT causes localized microvascular damage that starves the tumor and stimulates an acute inflammatory response, activating both innate and adaptive anti-tumor immunity [8,19]. Despite its high efficacy, conventional PDT (cPDT) faces clinical limitations, primarily procedure-related pain during illumination and restricted light penetration into thicker, hyperkeratotic, or heavily pigmented lesions [20]. To circumvent these challenges, the field has seen significant therapeutic innovation. A major advancement is daylight PDT (dPDT), which utilizes natural sunlight to continuously and gradually activate PpIX. This approach almost entirely eliminates the pain associated with cPDT while maintaining comparable efficacy for non-hyperkeratotic AKs and broad field cancerization. Additionally, to enhance drug delivery and tackle deeper or more resistant NMSCs, researchers are successfully employing physical pre-treatments such as microneedling, curettage, and ablative fractional lasers (AFXLs) [13]. The development of nanoparticle-based drug delivery systems and third-generation photosensitizers also promises to further enhance tumor targeting and overcome the constraints of tissue hypoxia and light penetration. Recent bibliometric analyses illustrate an explosive and continuous growth in PDT research over the last few decades, reflecting its transition from an experimental concept to a cornerstone of clinical practice. Given this vast and dynamic landscape, a focused and updated synthesis of the literature is essential. Therefore, the objective of this narrative review is to comprehensively evaluate the current role of PDT exclusively in the management of keratinocytic precancerous lesions and non-melanoma skin cancers. The discussion will specifically target actinic keratosis, field cancerization, actinic cheilitis, BCC (both superficial and nodular subtypes), Bowen’s disease (BD, SCC in situ), and invasive SCC. By synthesizing high-level clinical evidence, including randomized controlled trials and international guidelines, with emerging pre-clinical and technological innovations, this review aims to provide a structured overview of PDT’s evolving capabilities and limitations in modern dermatological oncology.

While several narrative and systematic reviews have previously addressed the applications of PDT in dermatological oncology, this work introduces a highly focused and innovative perspective designed to overcome key limitations in the existing literature. First, rather than presenting NMSCs as a broad, monolithic category, a common approach that often obscures critically different biological responses, this review provides a granular, histopathology-driven stratification of PDT efficacy. We systematically differentiate between highly responsive superficial lesions and more challenging subtypes, such as thick nodular, pigmented, and high-risk tumors, clearly outlining the biological boundaries of the treatment. Second, we bridge the gap between established protocols and the latest translational innovations, comprehensively analyzing novel physical and chemical optimization strategies, including epigenetically enhanced PDT (ePDT), 3D-printed microneedle delivery systems, and advanced nanotechnology. Lastly, this review offers a dedicated clinical synthesis of PDT’s crucial role in special patient populations, such as organ transplant recipients and Gorlin syndrome patients, culminating in an actionable synoptic recommendation matrix to serve as a direct, evidence-based reference for clinical decision-making.

## 2. Methods

We conducted a literature search on PubMed/MEDLINE, Embase, Scopus, the Cochrane Library, and ClinicalTrials.gov from January 2010 to January 2025, using keywords: “photodynamic therapy”, “Photosensitizing agents”, and “non-melanoma skin cancers”. Records were deduplicated across sources prior to screening. To minimize publication bias, reference lists of the included articles and relevant reviews were manually screened for additional eligible studies. The documents included narrative reviews, systematic reviews and meta-analyses, bibliometric analyses, consensus recommendations, randomized or controlled clinical studies, retrospective studies, case reports, Cochrane protocols, preclinical studies, and technical or methodological papers. All articles were screened for relevance to keratinocytic precancerous lesions and NMSC. Articles directly addressing AKs, field cancerization, BCC, Bowen disease (BD), cSCC, or NMSC were used as the core evidence base. Articles focused primarily on melanoma, cutaneous lymphoma, melanoma prognostic biomarkers, photothermal therapy (PTT), photoimmunotherapy, or photoacoustic laser killing were not used to infer efficacy for keratinocytic tumors; they were considered only when they contained directly relevant mechanistic, delivery, or scope-defining information, or when their inclusion clarified why conclusions should not be extended beyond the selected histotypes. The level of evidence was interpreted according to study design. Systematic reviews, meta-analyses, randomized trials, and consensus processes were considered higher-level clinical evidence when they directly addressed the selected conditions. Narrative reviews, bibliometric analyses, retrospective series, case reports, and preclinical studies were used descriptively and not treated as equivalent to comparative clinical evidence. When a topic was present only in review-level literature, this is stated. When a topic was not meaningfully addressed in the reviewed literature, the review states that this aspect was not addressed in the reviewed literature.

## 3. Mechanistic Basis Relevant to Keratinocytic Tumors

**Figure 1:**
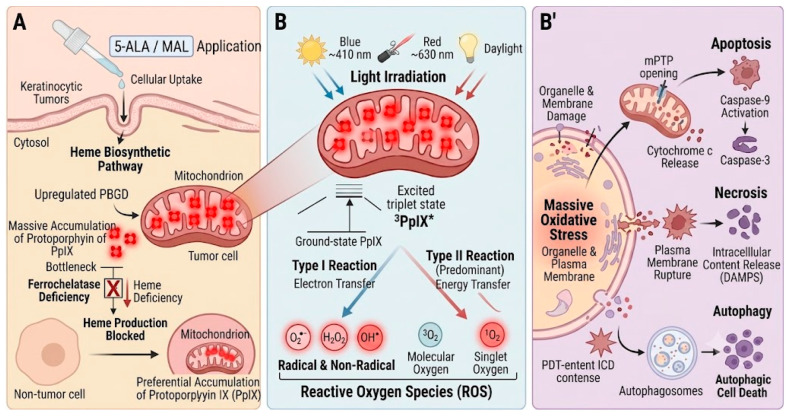
Schematic representation of the molecular, photochemical, and cellular cascade of ALA/MAL-mediated photodynamic therapy (PDT) in keratinocytic tumors. (**A**) Biosynthetic phase: Topical application of the prodrugs (5-ALA/MAL) leads to their enzymatic conversion to protoporphyrin IX (PpIX) within the mitochondria. In tumor cells, the upregulation of the PBGD enzyme and the concomitant deficiency in ferrochelatase create a “bottleneck” that causes a massive and selective accumulation of PpIX compared with healthy cells. (**B**) Photochemical Phase: Irradiation with light (blue, red, or daylight) excites the accumulated PpIX to its triplet state (^3^PpIX*) . Through Type I (electron transfer) and Type II (energy transfer, predominant) reactions, Reactive Oxygen Species (ROS) are generated, including radical species ($O_{2}^{\cdot -}$, $OH^{\cdot }$), non-radical peroxides ($H_{2}O_{2}$), and highly cytotoxic singlet oxygen (O21). (**B’**) Cell Death Phase: Massive oxidative stress causes damage to organelles and membranes, triggering three main pathways of cell death: apoptosis (mediated by the release of cytochrome c and activation of caspases), necrosis (rupture of the plasma membrane with the release of DAMPs), and autophagic cell death.

The fundamental basis of PDT relies on the synergistic interaction of three essential, individually non-toxic components: a photosensitizer (PS) or its metabolic precursor, a light source of an appropriate wavelength, and tissue molecular oxygen. The integration of these elements initiates a cascade of photochemical and photobiological reactions that culminate in targeted tumor destruction [21]. Please refer to Figure 1.

### 3.1. The Porphyrin Pathway and Protoporphyrin IX Accumulation

In the context of keratinocytic tumors, the most frequently employed agents are the prodrugs 5-aminolevulinic acid (5-ALA) and its lipophilic ester, methyl aminolevulinate (MAL) [11,15]. Once applied topically, these precursors bypass physiological feedback mechanisms and enter the heme biosynthetic pathway [10]. Within the cytosol and mitochondria, 5-ALA is metabolized into protoporphyrin IX (PpIX), a highly fluorescent and photoactive endogenous photosensitizer [22,23]. While the final enzymatic steps of PpIX synthesis occur within the mitochondria, its highly lipophilic nature causes it to rapidly redistribute and associate with intracellular lipid-rich membranes, primarily those of the endoplasmic reticulum (ER), outer mitochondrial membranes, and lysosomes, rather than remaining strictly confined to a single organelle [24,25,26]. This broad lipid-membrane localization pattern is a defining hallmark of porphyrin-mediated photosensitization, dictating the primary sites of subsequent photodamage [26]. Neoplastic cells, including those in BCC, SCC, and field cancerization, exhibit an altered enzymatic profile, specifically an upregulation of porphobilinogen deaminase and a relative deficiency in ferrochelatase, which impedes the conversion of PpIX to heme [24]. This enzymatic bottleneck, coupled with the altered stratum corneum and rapid proliferation of tumor cells, leads to a highly selective, transient accumulation of PpIX within the mitochondria and endoplasmic reticulum of malignant keratinocytes [24,25]. The lipophilicity of MAL allows for deeper tissue penetration compared to the hydrophilic 5-ALA, although both are highly effective in inducing intracellular PpIX aggregation [10]. Various physical and chemical enhancements, such as microneedling, fractional ablative lasers, and iron chelators (e.g., CP94 or desferrioxamine), can further optimize this accumulation, preventing the conversion of PpIX into non-photoactive components [7,24].

### 3.2. Photochemical Reactions: Type I and Type II Pathways

Upon light irradiation, the accumulated intracellular PpIX transitions to its excited triplet state (^3^PpIX*), initiating localized photochemical cascades. In the context of keratinocytic neoplasias, the clinical efficacy of these pathways is strictly governed by the optical and physiological microenvironment of the lesions [27,28]. Through intersystem crossing, the PS enters a relatively long-lived excited triplet state, which can react with surrounding molecules via two distinct photochemical pathways [29]. Under normoxic conditions, Type II energy transfer to ground-state molecular oxygen ($O_{2}$) yields highly cytotoxic singlet oxygen (O21), which is the primary driver of tumor destruction in superficial lesions like Bowen’s disease and thin actinic keratoses (AKs). However, fast-growing, hypermetabolic NMSC cells, particularly within the dense tumor nests of nodular BCCs, frequently exhibit localized tissue hypoxia. During continuous high-intensity conventional PDT (cPDT), the rapid depletion of local oxygen outpaces physiological blood perfusion, causing a transient shift toward Type I electron-transfer reactions. Type I reactions generate radical species (such as superoxide anions $O_{2}^{\cdot -}$ and hydroxyl radicals ${\mathrm{OH}}^{\cdot }$) that are less oxygen-dependent but carry a shorter therapeutic radius [23,30]. Type II reactions are generally considered the predominant mechanism of cytotoxicity in dermatological PDT, although both pathways occur simultaneously and their ratio depends strongly on the specific PS used and the oxygen concentration in the tumor microenvironment. Furthermore, the spatial propagation of these photochemical pathways is physically restricted by the optical properties of the lesions. In highly hyperkeratotic AKs and cutaneous squamous cell carcinomas, cSCCs, the thickened stratum corneum scatters and attenuates blue light (~410 nm), confining photochemical activation to the topmost epidermal layers. Conversely, red light (~630 nm) penetrates deeper but is heavily absorbed by melanin in pigmented BCC subtypes, where melanin not only acts as an optical shield but also functions as a potent intracellular antioxidant, scavenging both O21 and Type I free radicals. This biological bottleneck underlines the absolute necessity of light fractionation protocols (allowing tissue re-oxygenation for Type II reactions) and physical debulking to optimize the photochemical yield within NMSC tissue [29].

### 3.3. Mechanisms of Cell Death: Apoptosis, Necrosis, and Autophagy

Malignant keratinocytes in AKs, cSCCs, and BCCs possess highly efficient intrinsic survival mechanisms that render them resistant to standard DNA-damaging therapies [8,21]. PpIX-mediated PDT is uniquely suited to overcome these barriers due to its organelle-specific localization and oxygen-radical-mediated cytotoxicity, which bypasses classical genomic checkpoints [18,31]. A hallmark of UV-induced carcinogenesis in AKs and cSCCs is the presence of “UV-signature” mutations in the TP53 gene, which impair p53-dependent apoptotic pathways and lead to treatment resistance [32]. Because topically generated PpIX localizes preferentially within intracellular lipid membranes, particularly those of the ER and mitochondria, rather than the cell nucleus, the resulting ROS generation triggers a rapid, p53-independent apoptotic cascade [26,31,33]. Photodamage to these vital organellar membranes induces the direct opening of the mitochondrial permeability transition pore (mPTP) and mitochondrial outer membrane permeabilization, causing the release of cytochrome c and activating Caspases-9 and -3, completely bypassing the mutated p53 checkpoint [31,33]. In Basal Cell Carcinomas (BCCs), apoptosis is often physiologically blocked by the characteristic overexpression of the anti-apoptotic oncogene Bcl-2 [17]. Crucially, mitochondrial-localized PDT directly targets and photo-destroys Bcl-2 proteins during the initial phases of light activation, neutralizing this survival signal and facilitating rapid caspase activation [34]. When NMSC cells are exposed to high concentrations of photosensitizer and light, the mechanism shifts from programmed apoptosis to rapid necrosis, resulting in plasma membrane rupture [31,35]. In the chronically UV-damaged microenvironment of field cancerization (which is often highly immunosuppressed), PDT-induced necrosis is therapeutic: it triggers Immunogenic Cell Death (ICD) [31]. The physical disruption of the tumor cell membrane releases Damage-Associated Molecular Patterns (DAMPs), such as calreticulin exposure on the cell surface, and causes the extracellular release of HMGB1 and heat shock proteins (HSP70/90) [14,31]. These molecular signals alert resident antigen-presenting cells, reversing the local immune evasion and promoting the clearance of subclinical dysplastic clones [14,31]. Lastly, autophagy is initiated as an adaptive survival response by NMSC cells attempting to clear photodamaged organelles; however, excessive and irreversible photo-oxidative stress on the ER and lysosomal membranes eventually subverts this mechanism, driving the cell toward autophagic cell death [31,36].

### 3.4. Immune Response and Tumor Microenvironment Alterations

Beyond direct cellular cytotoxicity, PDT exerts its antitumoral effects through severe microvascular damage and the induction of a robust immune response [37]. The ROS generated during illumination rapidly damage the endothelial cells of the tumor vasculature, leading to thrombosis, vascular occlusion, and subsequent tumor starvation and hypoxia [31,38]. Simultaneously, the localized tissue damage and necrotic debris provoke an acute inflammatory response characterized by the rapid infiltration of neutrophils, macrophages, and mast cells into the treated area. PDT-induced cell death is highly immunogenic (Immunogenic Cell Death, ICD) [14]. The treatment induces the emission of Damage-Associated Molecular Patterns (DAMPs), including heat shock proteins (HSP70, HSP90), calreticulin, and HMGB1, which are recognized by Toll-like receptors (TLRs) on antigen-presenting cells. This triggers the maturation of dendritic cells, which then migrate to regional lymph nodes to present tumor-specific antigens, activating cytotoxic CD8+ T-cells and Natural Killer (NK) cells [31]. This transition from an innate acute inflammatory response to a targeted adaptive immune response is crucial not only for eradicating the primary lesion but also for controlling subclinical dysplasia within the concept of “field cancerization” [28,39].

### 3.5. Translation to Diverse Clinical Outcomes and Technological Advances

These fundamental mechanisms dictate the success of PDT across a wide spectrum of cutaneous and non-cutaneous malignancies [1,19,38]. The principles of ROS generation and selective cytotoxicity discussed above form the biological basis for treating AKs [40,41,42], Bowen’s disease (BD) [43], and BCC [44]. Furthermore, the mechanistic hurdles of PDT, such as poor light penetration through melanin and tissue hypoxia, have driven the development of advanced modalities. These include fractionated illumination, integration with ablative fractional lasers [45], the use of systemic extracorporeal photopheresis (ECP) for lymphomas [22], and the synthesis of third-generation nanocarriers, liposomes, and metal–organic frameworks (MOFs) engineered to overcome tumor resistance in aggressive forms like melanoma and invasive SCC [46,47,48,49].

## 4. Components and Protocols of Dermatological PDT

The clinical efficacy of PDT is highly dependent on the precise orchestration of its core protocols, which have evolved significantly to meet the needs of diverse dermatological oncology patients [1]. As highlighted by numerous bibliometric analyses, consensus guidelines, and systematic reviews, the treatment landscape requires a customized approach based on lesion type, location, and the patient’s specific immune and histological profile, encompassing everything from AKs and NMSC to cutaneous lymphomas [50].

### 4.1. Topical Photosensitizers and Drug Delivery Innovations in Dermatological PDT

#### 4.1.1. Aminolevulinic Acid Derivatives and Nanodelivery Systems

The foundational step in dermatological PDT is the topical application of a PS or its metabolic precursor [11,15]. In standard clinical practice, the hydrophilic prodrug 5-ALA and its more lipophilic ester, methyl aminolevulinate (MAL), are the primary agents used [11,15]. While both are highly effective, the lipid-rich stratum corneum poses a formidable barrier to their passive diffusion, often leading to heterogeneous drug distribution and limited depth of penetration (typically <2 mm), particularly in hyperkeratotic AKs or deeper nBCCs [13,20,41]. To bypass these pharmacological limits and enhance clinical efficacy, nanomedicine has actively reshaped photosensitizer formulation [28,31]. A key clinical milestone is the development of nanoemulsion-based drug delivery systems [4]. The commercially approved formulation BF-200 ALA stabilizes 5-ALA within a nanoemulsion, which significantly enhances epidermal penetration and metabolic conversion to PpIX [4]. Comparative trials demonstrate that BF-200 ALA achieves superior or non-inferior clearance rates for sBCC and AKs compared to conventional MAL [4,9]. Beyond nanoemulsions, highly advanced nanocarriers are being developed to optimize drug delivery, control release kinetics, and target the tumor microenvironment [28,47,51]. Liposomes, polymeric micelles, and hydrogels are widely investigated to encapsulate both hydrophilic and hydrophobic photosensitizers [47,51]. These systems protect the PS from premature degradation, increase aqueous solubility, and leverage the enhanced permeability and retention (EPR) effect to preferentially accumulate within malignant tissues [28,35]. For example, attachable hydrogels containing Indocyanine Green (ICG) have been developed for targeted near-infrared photothermal and photodynamic ablation of NMSCs [47,49]. Furthermore, inorganic nanoparticles, such as gold nanoparticles (AuNPs), are engineered as hybrid systems that actively target tumor-specific receptors (e.g., via epidermal growth factor receptor targeting) to amplify local reactive oxygen species (ROS) production [28,49]. Among next-generation, purely preclinical platforms, biodegradable Metal–Organic Frameworks (MOFs) and porphyrin-based nanoparticles synthesized via co-precipitation are being investigated as experimental “fourth-generation” photosensitizers [29,52]. These highly porous nanostructures can carry high payloads of active agents (including chlorins or porphyrins) and are engineered to integrate diagnostic imaging with precise therapeutic activation [29,52]. However, it must be explicitly emphasized that MOFs are currently far from any approved clinical utilization, and their investigation remains strictly confined to in vitro and in vivo animal models. Additionally, to bypass the stratum corneum painlessly and deliver these nanocarriers directly into deep tumor beds, researchers are utilizing Dissolving Microneedles (DMNs) and 3D-printed microneedle patches [39,51]. A notable preclinical strategy involves DMNs loaded with solid lipid nanoparticles (SLNs) encapsulating both paclitaxel and the near-infrared photosensitizer IR-780, facilitating a spatiotemporally controlled, synergistic chemo-photothermal and photodynamic attack against aggressive skin tumors [51]. Even biological nanocarriers, such as plant virus-based nanoparticles (e.g., tobacco mosaic virus), are being investigated to deliver photosensitizing payloads while simultaneously triggering systemic anti-tumor immunity [29,31]. The key nanodelivery systems, their mechanisms, and their target clinical applications are summarized in Table 1.

#### 4.1.2. Chlorin-Based Photosensitizers in NMSC PDT

While porphyrin precursors (5-ALA and MAL) dominate topical outpatient dermatological PDT due to their high selectivity, they are physically limited by shallow light activation wavelengths and the enzymatic constraints of the endogenous heme biosynthetic pathway [11,24]. To overcome these limitations, second-generation photosensitizers, specifically chlorin-type agents such as Temoporfin (mTHPC/Foscan), Talaporfin sodium, Radachlorin, and Fotolon, have been widely and successfully utilized in clinical oncology for over twenty years. These semisynthetic macrocycles, structurally derived from chlorophyll, are characterized by intense absorption bands in the red and near-infrared (NIR) spectrum (typically ranging from 650 to 670 nm) [53,54]. This red-shifted absorption dramatically increases the effective tissue penetration depth compared to standard porphyrin precursors, allowing light to reach deeper-seated dermal tumor structures [17,54]. In dermato-oncology, chlorin-based PDT has demonstrated excellent clinical outcomes, particularly in anatomically challenging and high-risk zones. For instance, Nadkernichnaya et al. reported highly effective complete clearance rates when utilizing chlorin-based PDT for basal cell carcinoma (BCC) localized in the critical H-zone of the face, a region where tissue-sparing therapy is crucial [55]. Similarly, the clinical use of systemic and intralesional Radachlorin and Fotolon has been extensively shown to offer superior photodynamic action in localized skin malignancies, combining swift selective accumulation with rapid systemic clearance, which significantly reduces the duration of post-treatment skin photosensitivity [56]. From a medicinal chemistry and translational perspective, the chlorin scaffold serves as a highly versatile platform for targeted delivery and combination regimens. Recent breakthroughs include the development of novel chlorin-e6 metallocomplex conjugates (utilizing Zinc, Indium, or Palladium) coupled with EGFR-targeting ligands, which demonstrate nanomolar-range phototoxicity against tumor cells, a rare capability for conventional photosensitizers [57]. Furthermore, Temoporfin (mTHPC) remains one of the most photodynamically active clinical agents available; a comprehensive decadal analysis highlights its extensive pre-clinical and clinical application in oncology, including the formulation of liposomal carriers that further refine its distribution and target delivery [54]. Ultimately, as thoroughly detailed in the seminal book by Reshetnikov and Mead, ‘The Medicine of Light,’ chlorin-based PDT represents a foundational pillar of modern photomedicine, bridging the gap between superficial tissue surface clearance and the deep-seated eradication of complex localized malignancies [58].

#### 4.1.3. Next-Generation Photosensitizers

To achieve deeper tissue penetration and absolute tumor selectivity, clinical research is transitioning from established second-generation agents, such as Silicon Phthalocyanine 4 (Pc 4), which has a long history of efficacy in NMSC and cutaneous T-cell lymphoma (CTCL) clinical trials [59], toward third-generation photosensitizers. Third-generation agents are defined by the chemical conjugation of established second-generation photosensitizing scaffolds (like chlorins or porphyrins) to tumor-homing biomolecules, including monoclonal antibodies, specific peptides (e.g., EGFR-targeting ligands), or folic acid, to achieve active molecular targeting and minimize off-target phototoxicity. This molecular evolution, combined with the nanocarriers discussed above, represents the true translational frontier of photodynamic dermato-oncology [52].

### 4.2. Light Sources and Dosimetry in cPDT

In cPDT, the choice of light source directly influences both tissue penetration and patient tolerability [30]. Red light (typically around 630 nm) provides deeper dermal penetration and is standardly indicated for thicker lesions like nodular BCCs, while blue light (~410 nm) is highly effective for activating superficially accumulated protoporphyrin IX (PpIX) in AKs, though continuous illumination is often associated with intense, sometimes dose-limiting pain [37,60]. To optimize dosimetry and minimize patient discomfort, modifications such as light fractionation (e.g., two-fold illumination schemes with a dark interval) are clinically employed to allow tissue re-oxygenation and prevent the rapid depletion of local oxygen, which is essential for Type II photochemical reactions [24,48]. While highly experimental approaches like two-photon excitation have been proposed in laboratory settings to achieve precise spatial activation, these techniques remain strictly restricted to basic research and are entirely inappropriate for clinical translation due to the prohibitive cost of the required ultra-fast laser systems and their lack of therapeutic scalability.

### 4.3. The Paradigm Shift: dPDT

To circumvent the primary adverse event of cPDT, procedure-related pain, dPDT has emerged as a transformative protocol, particularly for broad field cancerization and multiple Aks [41]. Rather than delivering a high-intensity dose of artificial light over a short period, dPDT utilizes natural sunlight (or artificial daylight simulators) to continuously and gradually activate PpIX as it is synthesized in the target cells [11]. By preventing the massive, sudden accumulation of PpIX and the subsequent intense inflammatory spike, dPDT achieves an almost painless experience while maintaining equivalent efficacy to cPDT for grade I and II non-hyperkeratotic AKs. The convenience, superior cosmetic outcomes, and high patient satisfaction have solidified dPDT as a cornerstone in the modern management of keratinocytic precancers, establishing it as a highly recommended protocol across global dermatological practices [61].

## 5. Clinical Efficacy by Target Pathology

The clinical efficacy of PDT varies significantly depending on the histopathology, thickness, and pigmentation of the target lesion. While the term NMSC encompasses a broad umbrella of malignancies, clinical evidence robustly supports the use of PDT primarily for superficial keratinocyte-derived neoplasias.

### 5.1. AK and Field Cancerization

AKs are the most common neoplastic lesions and are universally recognized as precursors to invasive cSCC [9]. Because it is impossible to predict which specific AK lesion will undergo malignant transformation, modern dermatological guidelines emphasize the necessity of field-directed therapy to treat both visible lesions and subclinical dysplasia (field cancerization) [62]. PDT is highly efficacious in this setting. Meta-analyses comparing the widely used photosensitizers demonstrate that both MAL and the nanoemulsion formulation of BF-200 ALA yield high complete response (CR) rates, with BF-200 ALA showing a marginally higher clearance rate in some comparative trials [9]. A major paradigm shift in AK management is the widespread adoption of dPDT. Consensus guidelines from Europe and Australia confirm that dPDT with MAL is as effective as conventional PDT cPDT for treating non-hyperkeratotic (Olsen Grade I and II) AKs on the face and scalp, while being virtually painless [41]. By utilizing natural sunlight for continuous, gradual PpIX activation, dPDT avoids the intense pain associated with the rapid ROS generation of cPDT [27]. For optimal results, hyperkeratotic lesions must be pre-treated (e.g., with curettage or keratolytic agents like salicylic acid) prior to dPDT [27]. Furthermore, expert consensus groups, such as the Photodynamic therapy in Actinic Keratosis Treatment (PAKT) panel, strongly recommend cyclic, field-directed PDT as a highly effective chemopreventative strategy to delay or suppress the development of new cSCCs [62]. Other innovations to optimize tolerability include pre-treatment with adapalene gel or employing shortened incubation times (e.g., 1 h). However, a critical distinction must be made regarding the latter: while a 1 h abbreviated incubation significantly mitigates post-procedural erythema and stinging, its long-term efficacy on subclinical dysplasia and sustained field cancerization control remains controversial compared to standard 3 h regimens. Shorter incubation times may restrict homogeneous PpIX accumulation in deeper epidermal layers, potentially compromising the clearance of subclinical clones while successfully treating superficial clinical lesions [63].

### 5.2. BCC

BCC is the most prevalent skin cancer globally, and PDT is firmly established as a frontline treatment for low-risk variants [21]. However, accurate pre-treatment assessment of the tumor subtype and depth is critical, as PDT efficacy is generally limited to a tissue penetration depth of approximately 2 mm [4].

- Superficial BCC (sBCC): PDT achieves excellent complete response rates (ranging from 75% to over 90%) for sBCC [44]. While surgical excision may have a slightly lower long-term recurrence rate, PDT is frequently preferred due to its superior cosmetic outcomes and tissue-sparing nature, making it ideal for large or multiple lesions [2].
- Nodular BCC (nBCC): Treating thicker nodular lesions poses a therapeutic challenge. A large randomized controlled multicenter study by Christensen et al. [44] demonstrated that a simplified single-PDT regimen was significantly less effective than the standard, approved double-PDT regimen (two treatments one week apart) for sBCC and thin nBCC [44]. To enhance efficacy in nBCC, physical pre-treatments such as deep curettage, debulking, or ablative fractional CO_2_ lasers are strictly recommended to allow the photosensitizer and light to reach the tumor base, yielding clearance rates up to 95% [60,64].
- Pigmented BCC (pBCC): Melanin acts as a severe optical barrier and an intracellular antioxidant that scavenges PDT-induced ROS, drastically decreasing the treatment’s success rate [3]. Because melanin absorbs heavily in the visible spectrum and neutralizes the photodynamic effect, standard PDT often fails in pBCC [3]. Consequently, mechanical debulking or electro-curettage to physically remove the pigmentation prior to illumination is mandatory to achieve complete responses [3].
- Gorlin Syndrome (Nevoid BCC Syndrome): Patients with Gorlin syndrome develop multiple BCCs throughout their lifetime, making repeated surgical excisions highly disfiguring [60]. PDT serves as a crucial, non-scarring alternative. Clinical trials have shown that both blue-light and red-light PDT are highly effective in clearing superficial and thin nodular BCCs in this specific population, preserving the patient’s cosmetic appearance and psychological well-being [60].

### 5.3. Bowen’s Disease (SCC In Situ) and Erythroplasia of Queyrat

BD represents the in situ form of cutaneous SCC and carries a 3–5% risk of progressing to invasive carcinoma [15]. PDT has proven to be a highly efficacious treatment for BD, often outperforming cryotherapy and topical 5-fluorouracil in both clearance rates (up to 80–100%) and cosmetic outcomes [43]. It is particularly valuable for large lesions, multifocal disease, or tumors located in areas prone to poor wound healing, such as the lower extremities [2]. Additionally, PDT has shown promising results in treating Erythroplasia of Queyrat (SCC in situ of the mucous membranes, such as the glans penis), providing a tissue-sparing alternative in anatomically and functionally sensitive areas [30].

### 5.4. Invasive Cutaneous Squamous Cell Carcinoma (cSCC)

In stark contrast to its success in superficial lesions, the role of PDT in invasive cSCC remains heavily restricted. Because cSCC has a substantial risk of deep tissue invasion and metastasis, surgical excision with clear histological margins remains the absolute gold standard [59]. Currently, the use of PDT for invasive cSCC is generally reserved for palliation, for patients who are strictly non-surgical candidates, or as a neoadjuvant therapy designed to shrink tumor margins prior to surgery [8]. For large or multifocal cSCCs located in cosmetically sensitive areas like the face or lips, combining conservative surgery with adjuvant PDT has been reported to help preserve functional integrity [1]. In the preclinical realm, researchers are exploring innovative methods to overcome the resistance of invasive SCC to PDT, such as utilizing microneedles loaded with hypoxia-activated bioreductive drugs (like tirapazamine) to counter the hypoxic tumor microenvironment or employing modified ALA-PDT protocols designed to trigger pyroptosis (a highly inflammatory form of programmed cell death) via the ROS-JNK-NLRP3 signaling pathway. However, these approaches remain experimental, and PDT cannot currently be recommended as a standalone curative treatment for invasive cSCC [50].

## 6. Optimization Strategies and Combined Therapies

While PDT is highly effective for superficial lesions, its efficacy is historically limited by the stratum corneum’s barrier function, limited light penetration depth (typically < 2 mm), tissue hypoxia, and optical barriers such as melanin [4,21]. To overcome these challenges and treat thicker NMSCs and resistant field cancerization, researchers have developed robust optimization strategies ranging from mechanical and chemical pre-treatments to advanced nanotechnology.

### 6.1. Physical Pre-Treatments and Tissue Preparation

The physical disruption of the skin barrier is paramount for facilitating the deep and homogenous penetration of topical PSs like 5-ALA and MAL [41].

- Curettage and Debulking: Mechanical preparation via curettage or surgical debulking is a fundamental step strongly recommended before PDT, particularly for nBCC, pBCC, and AKs [41]. By physically removing overlying scales, crusts, and melanin, clinicians significantly enhance CR rates while maintaining superior cosmetic outcomes [3].
- Ablative Fractional Lasers (AFXLs): The integration of AFXLs (such as CO_2_ and Er:YAG lasers) with PDT represents a major clinical breakthrough [64]. AFXL creates vertical microscopic ablation zones (micro-channels) in the skin, allowing the PS to bypass the stratum corneum and reach the deeper dermal layers where tumor nests reside [13]. Clinical trials demonstrate that AFXL-PDT yields significantly higher clearance rates for thick nBCCs and recurrent lesions compared to conventional PDT alone, with excellent long-term cosmetic results and low recurrence [7].
- Microneedling and Microdermabrasion: For less aggressive disruption, microneedling (e.g., using a dermaroller) creates transient micro-pores that boost drug delivery [45]. This technique is utilized not only for AKs but has also shown promise in enhancing PDT for primary cutaneous B-cell lymphomas (CBCLs) and cutaneous T-cell lymphomas (CTCLs) [25]. Similarly, standardized tape stripping and microdermabrasion effectively thin the stratum corneum, ensuring uniform PS distribution [62].
- Temperature Modulation: Elevating the local tissue temperature (hyperthermia) during the PS incubation period using heating pads has been shown to accelerate the enzymatic conversion of prodrugs into PpIX, yielding stronger phototoxic reactions and improved clearance [62].

### 6.2. Chemical Enhancers and Sequential Therapies

Beyond physical modalities, combining PDT with topical chemical agents can synergistically improve outcomes.

- Keratolytics and Retinoids: Pre-treating lesions with keratolytics (like urea or salicylic acid) or topical retinoids (such as adapalene gel) for several days prior to PDT thins the epidermis and promotes PpIX accumulation. Studies show that adapalene pre-treatment significantly reduces AK lesion counts compared to standard PDT [27].
- Sequential Chemotherapeutics: Utilizing topical 5-FU or imiquimod prior to or sequentially with PDT enhances the immune response and cellular destruction in widespread field cancerization.
- Iron Chelators: A novel strategy to boost intracellular PpIX involves the use of iron chelators, such as desferrioxamine (DFO) and CP94. Because ferrochelatase requires iron to convert PpIX into photo-inactive heme, chelating the available iron halts this conversion, driving massive PpIX accumulation and increasing targeted apoptosis [5,27].
- ePDT: To overcome the resistance of certain tumor cells to apoptosis (such as in invasive SCC or CTCL), researchers have introduced ePDT. Pre-treating cells with low-dose methotrexate epigenetically upregulates death receptors (e.g., FAS, DR4) and their ligands (FASL, TRAIL), drastically enhancing the extrinsic apoptotic pathway upon light illumination [65].

## 7. Special Populations and Specific Clinical Scenarios

While PDT is a standard non-invasive modality for the general population, it holds unique and indispensable value for specific cohorts of patients. For individuals with genetic predispositions to skin cancer or those suffering from chronic immunosuppression, repeated surgical excisions can lead to unacceptable morbidity, severe disfigurement, and exhaustion of surgical options. In these scenarios, the field-directed, repeatable, and tissue-sparing nature of PDT becomes a primary therapeutic strategy.

### 7.1. Organ Transplant Recipients (OTRs) and Immunocompromised Patients

Patients who have undergone solid organ transplantation (SOTRs) require long-term immunosuppressive therapy, which, combined with cumulative UV exposure, drastically increases their risk of developing NMSC. The standardized incidence ratio for cSCC and in situ carcinomas (such as BD) is estimated to be 65 to 250 times higher in transplant recipients than in the general population [15]. Furthermore, cSCCs in OTRs tend to be more aggressive, multi-focal, and carry a higher risk of metastasis [6]. Because OTRs frequently present with widespread field cancerization and hyperkeratotic actinic keratoses (AKs) that have high recurrence rates, conventional lesion-directed therapies are often insufficient [2,62]. Recent Delphi consensus panels strongly recommend the use of field-directed PDT as a chemopreventive measure to mitigate AKs and significantly reduce the risk of progression to invasive cSCC [62]. Clinical evidence strongly supports this prophylactic approach: cyclic PDT treatments (e.g., performed every 2 to 6 months over several years) have been shown to significantly delay the onset of new lesions and reduce the overall SCC lesion count by up to 95% at 24 months post-treatment [2]. Despite the clear benefits, anecdotal evidence and some clinical studies suggest that chronic immunosuppression may blunt the local inflammatory response necessary for optimal PDT clearance, sometimes resulting in lower CR rates compared to immunocompetent individuals [2,6,62]. To counteract this and maximize efficacy, experts recommend pre-treating the cancerized fields in OTRs with keratolytics, ablative fractional lasers (AFLs), or microdermabrasion prior to PS application [62]. Additionally, because OTRs may require treatment over large body surface areas and frequently report lower pain thresholds, modalities like dPDT or AFL-assisted dPDT are highly recommended to provide a virtually painless experience while maintaining effective disease control [6].

### 7.2. Gorlin Syndrome (Nevoid BCC Syndrome)

Gorlin syndrome, or Basal Cell Nevus Syndrome (BCNS), is a rare autosomal dominant genetic disorder caused by mutations in the PTCH-1 gene of the hedgehog signaling pathway [60]. Patients with this syndrome develop multiple BCCs starting as early as childhood or adolescence, often numbering in the hundreds over a patient’s lifetime [60]. Because radiotherapy is strictly contraindicated in these patients, as it paradoxically triggers the rapid development of new BCCs, and repeated surgeries lead to extensive, debilitating scarring, PDT provides a crucial non-surgical, non-scarring alternative [17]. Topical PDT utilizing ALA or MAL is highly effective for clearing superficial and thin nodular BCCs in Gorlin syndrome patients, allowing clinicians to treat wide fields of tumors simultaneously [17]. A notable bilaterally controlled trial comparing blue light (400 nm) versus red light (635 nm) ALA-PDT in BCNS patients (treating over 140 BCCs) demonstrated that blue light was non-inferior to red light, achieving remarkable clearance rates of 98% and 93%, respectively, with excellent cosmetic outcomes [60]. For thicker or more resistant nodular lesions in this population, combination therapies are frequently employed. Pre-treating the tumors with fully ablative CO_2_ lasers to safely debulk the visible abnormal tissue immediately prior to PDT significantly improves the PS penetration to the tumor base, yielding excellent long-term success rates [64]. In exceptionally challenging or widespread cases, systemic PDT using intravenous PS (such as Photofrin) combined with interstitial optic diffusers has also demonstrated efficacy, though topical applications remain the preferred frontline choice to avoid prolonged systemic photosensitivity [2]. Ultimately, the integration of PDT into the lifelong management of Gorlin syndrome drastically reduces the need for surgical interventions, thereby preserving the patient’s functional integrity and psychological well-being [17,60].

## 8. Safety Profile, Tolerability, and Cosmetic Outcomes

A critical factor driving the widespread adoption of PDT in dermatological oncology is its highly favorable safety profile and tissue-sparing nature. However, the management of procedure-related discomfort and local skin reactions remains an essential component of clinical practice to ensure patient adherence [42].

### 8.1. Pain Management and Patient Tolerability

The most significant dose-limiting adverse event in cPDT is pain, frequently described by patients as a severe burning or stinging sensation during light illumination [27]. To mitigate this and improve patient compliance, novel protocols have been aggressively investigated. dPDT has revolutionized the patient experience. By utilizing natural sunlight to continuously and gradually activate PpIX, dPDT prevents the sudden spike in ROS that causes acute nerve stimulation. As established by international consensus guidelines, dPDT is nearly painless and can be effectively performed year-round in suitable climates, provided patients use a chemical sunscreen (avoiding physical filters like zinc oxide that block visible light) [20,41]. Furthermore, researchers have developed “painless” cPDT protocols for use in the clinical setting. A notable approach involves the simultaneous application of 20% ALA and immediate illumination with a blue light source for 30 to 60 min. Bilaterally controlled trials demonstrate that this simultaneous regimen nearly eliminates pain (substantially reducing visual analog scale pain scores) while providing clinical efficacy and actinic keratosis AK lesion clearance rates equivalent to conventional protocols [63].

### 8.2. Expected Local Adverse Events

Following PDT, localized skin reactions are anticipated and generally serve as clinical indicators of a successful phototoxic and inflammatory response. Common and expected side effects include mild-to-moderate erythema, edema, localized pruritus, scaling, and crusting. Crucially, residual skin phototoxicity represents a major clinical side effect that demands rigorous patient education and compliance to prevent accidental phototoxic tissue damage [27]. These acute reactions typically peak within 48 to 72 h and resolve completely within a few days to weeks. To boost efficacy without drastically increasing side effects, physical pretreatments are frequently employed. Techniques such as microneedling (e.g., using a dermaroller) or thermo-mechanical fractional injury (TMFI), which creates micropores by evaporating tissue water in milliseconds, significantly enhance PS penetration [28,45]. While these pretreatments may slightly increase local erythema, they maintain an excellent overall safety profile with very low rates of severe complications like ulceration, secondary bacterial infection, or persistent dyschromia [27].

### 8.3. Cosmetic Outcomes and Scar Prevention

A major clinical advantage of PDT over traditional surgical excision or destructive modalities (such as cryotherapy) is its superior cosmetic outcome, a factor that heavily dictates patient preference [62]. Meta-analyses comparing treatments for superficial BCC and BD robustly demonstrate that PDT yields significantly higher rates of “good” or “excellent” cosmetic ratings compared to surgery and cryotherapy [15,50]. The selective nature of PDT ensures that surrounding healthy tissue is spared, minimizing scar formation. Standard evaluation criteria for PDT efficacy often highlight complete lesion regression leaving only intact skin with minimal hyper- or hypopigmentation [40]. Even when combining PDT with aggressive pretreatments, such as fully ablative CO_2_ lasers for thicker nodular BCCs, objective scar assessments using tools like the Vancouver Scar Scale (VSS) confirm that patients achieve highly satisfactory, long-term aesthetic results [44,64].

## 9. Discussion

The reviewed evidence across the provided literature supports a clear and distinct hierarchy of clinical maturity regarding the application of PDT in dermatological oncology. Rather than being a monolithic intervention, PDT represents a highly adaptable platform whose clinical success is intrinsically tied to the specific histopathology, thickness, pigmentation, and location of the target lesion (summarized in Table 2). At the pinnacle of this clinical hierarchy lie AK and field cancerization, which possess the broadest and most coherent evidence base. PDT is exceptionally attractive in this setting because it successfully addresses “field cancerization”, treating both clinically visible AKs and the surrounding subclinical photodamaged skin to delay or prevent the onset of invasive carcinomas [6]. Furthermore, a major obstacle to cPDT has historically been procedure-related pain, which can severely limit patient compliance. The widespread adoption of dPDT has revolutionized this aspect; by utilizing continuous, low-intensity natural sunlight, dPDT achieves non-inferior clearance rates for non-hyperkeratotic AKs compared to cPDT, while being virtually painless [6]. This innovation has cemented PDT’s role as a highly practical, patient-friendly option for widespread actinic damage. Moving down the hierarchy, superficial sBCC and BD (cSCC in situ) represent highly validated indications. In these superficial malignancies, PDT provides a tissue-sparing alternative to surgery. While systematic reviews confirm that surgical excision remains the gold standard for BCC overall due to offering the lowest long-term recurrence rates [27], PDT frequently yields demonstrably superior cosmetic outcomes. When considering patient preferences, the desire for excellent cosmesis and the avoidance of scarring often balance out the slightly higher risk of recurrence, particularly for large lesions, multiple tumors, or lesions in functionally sensitive areas [17]. However, the therapeutic reliability of conventional PDT wanes significantly when confronting thicker nBCC, pBCC and aggressive histologies [17]. In pigmented lesions, melanin acts as both an optical barrier that scatters light and an intracellular antioxidant that neutralizes the ROS generated by PDT, drastically decreasing the treatment’s success [3]. In these cases, mechanical or laser-assisted debulking and curettage prior to PS application are mandatory to improve outcomes [3]. Crucially, the discussion surrounding high-risk BCCs (e.g., infiltrative, morpheaform, or micronodular subtypes) and invasive cSCC reveals strict limitations. Evidence supporting PDT for invasive cSCC and aggressive BCC remains sparse, heterogeneous, and explicitly contraindicated as a standalone curative treatment due to the high risk of deep recurrence and metastasis associated with inadequate light and drug penetration [2,17]. Thus, while PDT excels in managing precancerous and superficial disease, it cannot currently replace surgical excision with margin control for invasive, high-risk malignancies.

## 10. Conclusions and Future Perspectives

PDT is strongly supported as a frontline, field-directed, and cosmetically superior treatment for AK, field cancerization, superficial or selected low-risk nodular BCC and BD. However, its application in thick nodular BCC, pigmented tumors, and invasive cSCC remains limited and requires cautious patient selection or adjunctive physical pretreatments. A paramount conclusion is the necessity of precise histopathological stratification in clinical practice. The continued use of NMSC as a broad umbrella category obscures critically important differences in tumor response, recurrence rates, and optimal protocols. Future guidelines and trials must analyze these entities separately to provide actionable recommendations. The future expansion of PDT relies on overcoming current physical and biochemical barriers. Immediate clinical advancements will likely focus on optimizing photosensitizer delivery through ablative fractional lasers (AFXLs) and microneedling. Looking further ahead, the integration of nanotechnology (e.g., targeted nanocarriers and liposomes) and photoimmunotherapy holds immense potential to enhance deep-tissue penetration, overcome hypoxic tumor microenvironments, and trigger systemic anti-tumor immunity. Until these innovations mature, PDT remains an indispensable, tissue-preserving tool in modern dermato-oncology, provided that careful patient and lesion selection is maintained.

## Figures and Tables

**Table 1:** Synoptic overview of nanodelivery systems for topical photosensitizers in dermatological PDT.

Nanodelivery System	Photosensitizer /Payload	Mechanism & Optimization Advantage	Target Pathology & Clinical Context
Nanoemulsion	5-Aminolevulinic Acid (BF-200 ALA)	Enhances chemical stability of 5-ALA; significantly improves epidermal penetration across the lipid-rich stratum corneum.	Actinic Keratosis (AK), Field Cancerization, superficial BCC (sBCC).
Liposomes/Polymeric Micelles	5-ALA, MAL, and hydrophobic photosensitizers	Protects cargo from degradation; improves aqueous solubility; exploits the EPR effect to accumulate in malignant tissue.	Non-Melanoma Skin Cancer (NMSC), cutaneous lymphomas, deep tumor nests.
Hydrogel Patches	Indocyanine Green (ICG)	Attachable, biocompatible patch that provides sustained release and localized photothermal/photodynamic synergy upon NIR irradiation.	Cutaneous melanoma, superficial skin cancers, localized NMSC.
Gold Nanoparticles (AuNPs)	Hybrid organic-inorganic photosensitizers	Enhances localized electromagnetic fields to boost ROS generation; can be conjugated with targeting ligands (e.g., anti-EGFR).	Aggressive skin malignancies, metastatic melanoma.
Metal–Organic Frameworks (MOFs)	Porphyrins, Chlorins, and Bacteriochlorins	Highly porous, biodegradable nanostructures; allows precise, targeted delivery of high payloads combined with diagnostic imaging.	Deep-seated NMSC, treatment-resistant skin cancers. Strictly experimental/Preclinical models (far from approved clinical translation).
Dissolving Microneedles (DMNs)	Solid Lipid Nanoparticles (SLNs) loaded with IR-780 & Chemotherapeutics	Painlessly punctures the stratum corneum to deliver SLNs directly into the tumor bed; provides spatiotemporal pulsatile release.	Cutaneous melanoma, thick nodular BCC, hyperkeratotic lesions.
Plant Virus-Based Nanocarriers	Porphyrin derivatives (e.g., TMV-based vectors)	Natural biomaterials that deliver photosensitizers deep into tissue while acting as an adjuvant to trigger robust systemic anti-tumor immunity.	Widespread skin malignancies, metastatic skin cancers.

**Table 2:** Synoptic matrix of clinical recommendations, protocol selection, and optimization strategies for photodynamic therapy (PDT) in keratinocytic lesions and non-melanoma skin cancer (NMSC).

Pathology & Clinical Scenario	Level of Clinical Support	Recommended Protocol	Optimization & Pre-Treatment Strategies	Major Clinical Caveats & Limitations
Actinic Keratosis (AK), Field Cancerization & Actinic Cheilitis	Highest/Frontline Strategy (Strong international consensus).	Daylight PDT (dPDT) with topically applied MAL for Olsen Grade I/II lesions. Conventional PDT (cPDT) (red/blue light) for specific single lesions.	Physical scale removal via curettage. Epidermal thinning using keratolytics (salicylic acid, urea) or topical retinoids (adapalene gel).	dPDT is restricted to non-hyperkeratotic lesions. Shortened incubation times (e.g., 1 h) reduce pain but may compromise deep field clearance of subclinical clones.
Superficial BCC (sBCC) & Bowen’s Disease (BD)	High/Validated Frontline Alternative.	(cPDT using the standard approved double-session regimen (two treatments spaced 1 week apart).	Implementation of light fractionation protocols. Sequential combination with topical 5-fluorouracil or imiquimod to boost regional cellular destruction.	Surgical excision provides lower long-term recurrence rates. PDT is selected primarily when cosmetic outcome, scar prevention, or tissue-sparing is a clinical priority.
Erythroplasia of Queyrat (SCC in situ of mucous membranes)	High/Targeted Indication.	Standard topical PDT modified for mucosal applications.	Careful protection of surrounding healthy tissue. Utilization of specialized tissue-sparing illumination profiles.	Limited to anatomically and functionally sensitive areas (e.g., glans penis) where surgery causes high morbidity.
Nodular BCC (nBCC)	Moderate (Moderate/restricted for standard topical ALA/MAL; High for chlorin-based PDT in larger or deep tumors).	1. Standard: Laser-assisted cPDT using red light (~630 nm) with topical ALA/MAL. 2. For Large/Thick Tumors: Chlorin-based cPDT (using Radachlorin, Fotolon, or Foscan) with deep-penetrating red/NIR light (650–670 nm).	Mandatory physical debulking, deep curettage, or AFXL pre-treatment (specifically for standard ALA/MAL to facilitate penetration). Intralesional or systemic administration of second-generation chlorin PSs to maximize deep-tissue drug accumulation in large masses.	Standard ALA/MAL efficacy drastically drops if tumor thickness exceeds the 2 mm optical penetration depth barrier. Chlorin-based PDT successfully treats larger/thicker tumors (including in the facial H-zone) with excellent complete clearance and scar-free cosmetic outcomes, but requires strict monitoring of residual phototoxicity.
Pigmented BCC (pBCC)	Low/Poor Response (Unless heavily optimized).	Modified cPDT protocols following aggressive physical preparation.	Vigorous mechanical debulking or electro-curettage to physically remove melanin. Preclinical/Experimental: Melanin-mediated multi-photon PDT using near-infrared (NIR) light.	Melanin acts as a severe optical barrier that scatters visible light. Melanin functions as an intracellular antioxidant, actively scavenging PDT-induced ROS.
Invasive Cutaneous SCC (cSCC)	Strictly Restricted/Limited.	Contraindicated as a standalone curative treatment.	Used strictly as a palliative intervention, for non-surgical candidates, or as a neoadjuvant therapy combined with conservative surgery to shrink margins.	High risk of deep tissue invasion and metastasis. Surgical excision with clear histological margins remains the absolute gold standard.
Organ Transplant Recipients (OTRs) (Immunocompromised Cohorts)	High/Strongly Recommended Prophylaxis.	Prophylactic, cyclic field-directed PDT (performed every 2 to 6 months over several years).	Aggressive skin preparation via keratolytics, microdermabrasion, or AFXL. Utilization of dPDT or AFL-assisted dPDT to manage lower pain thresholds.	Chronic immunosuppression can blunt the local inflammatory response, sometimes reducing complete clearance rates. Crucial for managing a 65 to 250 times higher risk of aggressive, multi-focal cSCC.
Gorlin Syndrome (Nevoid Basal Cell Carcinoma Syndrome)	High/Indispensable Maintenance Therapy.	Topical ALA-PDT using either blue light (400 nm) or red light (635 nm) (both show non-inferior clinical clearance).	Pre-treatment of nodular lesions with fully ablative CO_2_ lasers to safely debulk tissue. Advanced/Widespread cases: Systemic PDT (intravenous Photofrin) with interstitial optic diffusers.	Radiotherapy is strictly contraindicated in these patients as it triggers new primary tumors. Designed as a lifelong, non-scarring approach to minimize continuous surgical disfigurement.

## Data Availability

No new data were created or analyzed in this study. Data sharing is not applicable to this article.

## Abbreviations

The following abbreviations are used in this manuscript:

5-ALA	5-aminolevulinic acid
5-FU	5-fluorouracil
AFL/AFXL	Ablative Fractional Lasers
AK/AKs	Actinic Keratosis/Actinic Keratoses
AuNPs	Gold nanoparticles
BCC/BCCs	Basal Cell Carcinoma/Basal Cell Carcinomas
BCNS	Basal Cell Nevus Syndrome
BD	Bowen’s disease
CBCL	Cutaneous B-cell Lymphomas
cPDT	Conventional Photodynamic Therapy
CR	Complete Response
cSCC	Cutaneous Squamous Cell Carcinoma
DAMPs	Damage-Associated Molecular Patterns
dPDT	Daylight Photodynamic Therapy
ePDT	Epigenetically Enhanced Photodynamic Therapy
MAL	Methyl Aminolevulinate
MOFs	Metal–Organic Frameworks
mPTP	Mitochondrial Permeability Transition Pore
nBCC	Nodular Basal Cell Carcinoma
NIR	Near-Infrared
NK	Natural Killer cells
NMSC/NMSCs	Non-Melanoma Skin Cancer/Non-Melanoma Skin Cancers
OTRs	Organ Transplant Recipients
PAKT	Photodynamic therapy in Actinic Keratosis Treatment panel
pBCC	Pigmented Basal Cell Carcinoma
PBGD	Porphobilinogen Deaminase
PDT	Photodynamic Therapy
PpIX	Protoporphyrin IX
PTT	Photothermal Therapy
ROS	Reactive Oxygen Species
sBCC	Superficial Basal Cell Carcinoma
SCC	Squamous Cell Carcinoma
SLNs	Solid Lipid Nanoparticles
SOTRs	Solid Organ Transplant Recipients
TLRs	Toll-like Receptors
UV	Ultraviolet

## References

[1] Chen Q., Bai M., Lu M., Chen J., Wang S., Sun P., Li L., Cai H. (2025). Trends and Emerging Frontiers of Photodynamic Therapy for Non-Melanoma Skin Cancer (1979—October 2024): A Bibliometric Analysis. Front. Oncol..

[2] Griffin L.L., Lear J.T. (2016). Photodynamic Therapy and Non-Melanoma Skin Cancer. Cancers.

[3] Mazur E., Kwiatkowska D., Reich A. (2023). Photodynamic Therapy in Pigmented Basal Cell Carcinoma—A Review. Biomedicines.

[4] Mørk E., Mjønes P., Foss O.A., Mørk C., Bachmann I.M., Kroon S., Dotterud L.K., Helsing P., Vatne Ø., Christensen E. (2024). Clinical versus Histological Assessment of Basal Cell Carcinoma Subtype and Thickness of Tumours Selected for Photodynamic Therapy. Acta Derm. Venereol..

[5] Ceryn J., Lesiak A., Sobolewska-Sztychny D., Noweta M., Ciążyńska M., Narbutt J. (2025). Actinic Keratosis: Comprehensive Review of Current Treatments and Emerging Therapeutic Innovations. Postepy Dermatol. Alergol..

[6] Farberg A.S., Marson J.W., Soleymani T. (2023). Advances in Photodynamic Therapy for the Treatment of Actinic Keratosis and Nonmelanoma Skin Cancer: A Narrative Review. Dermatol. Ther..

[7] Alma A., Pongetti L., Clementi A., Chester J., Toccaceli M., Ciardo S., Zappia E., Manfredini M., Pellacani G., Greco M. (2023). Combined Carbon Dioxide Laser with Photodynamic Therapy for Nodular Basal Cell Carcinoma Monitored by Reflectance Confocal Microscopy. Medicina.

[8] Hua Y., Tian X., Zhang X., Song G., Liu Y., Zhao Y., Gao Y., Yin F. (2024). Applications and Challenges of Photodynamic Therapy in the Treatment of Skin Malignancies. Front. Pharmacol..

[9] Mpourazanis G., Konschake W., Vogiatzis R., Papalexis P., Georgakopoulou V.E., Ntritsos G., Sklapani P., Trakas N. (2022). The Role and Effectiveness of Photodynamic Therapy on Patients with Actinic Keratosis: A Systematic Review and Meta-Analysis. Cureus.

[10] Cohen D.K., Lee P.K. (2016). Photodynamic Therapy for Non-Melanoma Skin Cancers. Cancers.

[11] Balakirski G., Lehmann P., Szeimies R.-M., Hofmann S.C. (2024). Photodynamic Therapy in Dermatology: Established and New Indications. J. Dtsch. Dermatol. Ges..

[12] Galitzer B.I. (2021). Photodynamic Therapy for Actinic Keratoses of the Upper Extremities Using 10% Aminolevulinic Acid Gel, Red Light, and Adapalene Pretreatment. J. Clin. Aesthet. Dermatol..

[13] Champeau M., Vignoud S., Mortier L., Mordon S. (2019). Photodynamic Therapy for Skin Cancer: How to Enhance Drug Penetration?. J. Photochem. Photobiol. B.

[14] Dudzik T., Domański I., Makuch S. (2024). The Impact of Photodynamic Therapy on Immune System in Cancer—An Update. Front. Immunol..

[15] Antonetti P., Pellegrini C., Caponio C., Bruni M., Dragone L., Mastrangelo M., Esposito M., Fargnoli M.C. (2024). Photodynamic Therapy for the Treatment of Bowen’s Disease: A Review on Efficacy, Non-Invasive Treatment Monitoring, Tolerability, and Cosmetic Outcome. Biomedicines.

[16] Caccavale S., Tancredi V., Vitiello P., Sica A., Ronchi A., Franco R., Pastore F., Argenziano G. (2022). Photodynamic Therapy as an Effective Treatment for Cutaneous Lymphomas. Pharmaceutics.

[17] Collier N.J., Rhodes L.E. (2020). Photodynamic Therapy for Basal Cell Carcinoma: The Clinical Context for Future Research Priorities. Molecules.

[18] Naidoo C., Kruger C.A., Abrahamse H. (2018). Photodynamic Therapy for Metastatic Melanoma Treatment: A Review. Technol. Cancer Res. Treat..

[19] Zalewski A., Musiał W., Jankowska-Konsur A. (2025). Photodynamic Therapy in Primary Cutaneous Skin Lymphoma-Systematic Review. J. Clin. Med..

[20] Cantisani C., Paolino G., Pellacani G., Didona D., Scarno M., Faina V., Gobello T., Calvieri S. (2016). MAL Daylight Photodynamic Therapy for Actinic Keratosis: Clinical and Imaging Evaluation by 3D Camera. Int. J. Mol. Sci..

[21] Koumprentziotis I.-A., Rompoti N., Liopyris K., Nicolaidou E., Stratigos A. (2024). Photodynamic Therapy for the Treatment of Basal Cell Carcinoma: A Comprehensive Review of Randomized Controlled Trials. Dermatol. Pract. Concept..

[22] Christensen E., Foss O.A., Holien T., Juzenas P., Peng Q. (2024). Application of Photodynamic Therapy with 5-Aminolevulinic Acid to Extracorporeal Photopheresis in the Treatment of Cutaneous T-Cell Lymphoma: A First-in-Human Phase I/II Study. Pharmaceutics.

[23] Sun J., Zhao H., Fu L., Cui J., Yang Y. (2023). Global Trends and Research Progress of Photodynamic Therapy in Skin Cancer: A Bibliometric Analysis and Literature Review. Clin. Cosmet. Investig. Dermatol..

[24] de Souza A.L.R., Marra K., Gunn J., Samkoe K.S., Kanick S.C., Davis S.C., Chapman M.S., Maytin E.V., Hasan T., Pogue B.W. (2016). Comparing Desferrioxamine and Light Fractionation Enhancement of ALA-PpIX Photodynamic Therapy in Skin Cancer. Br. J. Cancer.

[25] Liu W.-T., Wang H.-T., Yeh Y.-H., Wong T.-W. (2023). An Update on Recent Advances of Photodynamic Therapy for Primary Cutaneous Lymphomas. Pharmaceutics.

[26] Agostinis P., Berg K., Cengel K.A., Foster T.H., Girotti A.W., Gollnick S.O., Hahn S.M., Hamblin M.R., Juzeniene A., Kessel D. (2011). Photodynamic Therapy of Cancer: An Update. CA Cancer J. Clin..

[27] Sotiriou E., Kiritsi D., Chaitidis N., Arabatzis M., Lallas A., Vakirlis E. (2025). Daylight Photodynamic Therapy for Actinic Keratosis and Field Cancerization: A Narrative Review. Cancers.

[28] Xu M., Kong L., Jamil M. (2024). Advancements in Skin Cancer Treatment: Focus on Photodynamic Therapy: A Review. Am. J. Cancer Res..

[29] Zou Y., Chen J., Qu Y., Luo X., Wang W., Zheng X. (2025). Evolution of nMOFs in Photodynamic Therapy: From Porphyrins to Chlorins and Bacteriochlorins for Better Efficacy. Front. Pharmacol..

[30] Tampa M., Sarbu M.-I., Matei C., Mitran C.-I., Mitran M.-I., Caruntu C., Constantin C., Neagu M., Georgescu S.-R. (2019). Photodynamic Therapy: A Hot Topic in Dermato-Oncology. Oncol. Lett..

[31] Falk-Mahapatra R., Gollnick S.O. (2020). Photodynamic Therapy and Immunity: An Update. Photochem. Photobiol..

[32] Brash D.E., Rudolph J.A., Simon J.A., Lin A., McKenna G.J., Baden H.P., Halperin A.J., Pontén J. (1991). A Role for Sunlight in Skin Cancer: UV-Induced P53 Mutations in Squamous Cell Carcinoma. Proc. Natl. Acad. Sci. USA.

[33] Tong Z., Singh G., Rainbow A.J. (2000). The Role of the P53 Tumor Suppressor in the Response of Human Cells to Photofrin-Mediated Photodynamic Therapy. Photochem. Photobiol..

[34] Xue L.Y., Chiu S.M., Oleinick N.L. (2001). Photochemical Destruction of the Bcl-2 Oncoprotein during Photodynamic Therapy with the Phthalocyanine Photosensitizer Pc 4. Oncogene.

[35] Nkune N.W., Abrahamse H. (2021). Nanoparticle-Based Drug Delivery Systems for Photodynamic Therapy of Metastatic Melanoma: A Review. Int. J. Mol. Sci..

[36] Hou Y.-J., Yang X.-X., Liu R.-Q., Zhao D., Guo C.-X., Zhu A.-C., Wen M.-N., Liu Z., Qu G.-F., Meng H.-X. (2020). Pathological Mechanism of Photodynamic Therapy and Photothermal Therapy Based on Nanoparticles. Int. J. Nanomed..

[37] Lowdell C.P., Ash D.V., Driver I., Brown S.B. (1993). Interstitial Photodynamic Therapy. Clinical Experience with Diffusing Fibres in the Treatment of Cutaneous and Subcutaneous Tumours. Br. J. Cancer.

[38] Kim T.E., Chang J.-E. (2023). Recent Studies in Photodynamic Therapy for Cancer Treatment: From Basic Research to Clinical Trials. Pharmaceutics.

[39] Obalola A.A., Abrahamse H., Dhilip Kumar S.S. (2025). 3D-Printed Biopolymer-Based Microneedle for Enhanced Photodynamic Therapy in Melanoma Treatment. Front. Oncol..

[40] Yi Q., Liu J. (2023). Safety of Photodynamic Therapy Combined with Surgical Excision in Patients with Actinic Keratosis and Risk Factors for Secondary Cutaneous Squamous Cell Carcinoma. Am. J. Transl. Res..

[41] See J.-A., Shumack S., Murrell D.F., Rubel D.M., Fernández-Peñas P., Salmon R., Hewitt D., Foley P., Spelman L. (2016). Consensus Recommendations on the Use of Daylight Photodynamic Therapy with Methyl Aminolevulinate Cream for Actinic Keratoses in Australia. Australas. J. Dermatol..

[42] Foley K., Gupta A.K., Martin G., Tweed J.A., Villanueva E., Carviel J. (2019). Topical Treatments and Photodynamic Therapy for Actinic Keratosis of the Face and Scalp. Cochrane Database Syst. Rev..

[43] Kang K., Bacci S. (2024). Photodynamic Therapy 2.0. Biomedicines.

[44] Christensen E., Mørk E., Foss O.A., Mørk C., Kroon S., Dotterud L.K., Helsing P., Vatne Ø., Skogvoll E., Mjønes P. (2024). New, Simplified versus Standard Photodynamic Therapy (PDT) Regimen for Superficial and Nodular Basal Cell Carcinoma (BCC): A Single-Blind, Non-Inferiority, Randomised Controlled Multicentre Study. PLoS ONE.

[45] Toulemonde E., Faiz S., Dubois R., Verhasselt-Crinquette M., Carpentier O., Abi Rached H., Mortier L. (2023). Photodynamic Therapy for the Treatment of Primary Cutaneous B-Cell Marginal Zone Lymphoma: A Series of 4 Patients. JAAD Case Rep..

[46] Ding L., Gosh A., Lee D.J., Emri G., Huss W.J., Bogner P.N., Paragh G. (2022). Prognostic Biomarkers of Cutaneous Melanoma. Photodermatol. Photoimmunol. Photomed..

[47] Hwang J., Jin J.-O. (2020). Attachable Hydrogel Containing Indocyanine Green for Selective Photothermal Therapy against Melanoma. Biomolecules.

[48] Pires L., Khattak S., Pratavieira S., Calcada C., Romano R., Yucel Y., Bagnato V.S., Kurachi C., Wilson B.C. (2024). Femtosecond Pulsed Laser Photodynamic Therapy Activates Melanin and Eradicates Malignant Melanoma. Proc. Natl. Acad. Sci. USA.

[49] Lopes J., Ferreira-Gonçalves T., Figueiredo I.V., Rodrigues C.M.P., Ferreira H., Ferreira D., Viana A.S., Faísca P., Gaspar M.M., Coelho J.M.P. (2021). Proof-of-Concept Study of Multifunctional Hybrid Nanoparticle System Combined with NIR Laser Irradiation for the Treatment of Melanoma. Biomolecules.

[50] Ou-Yang Y., Zheng Y., Mills K.E. (2023). Photodynamic Therapy for Skin Carcinomas: A Systematic Review and Meta-Analysis. Front. Med..

[51] Qin W., Quan G., Sun Y., Chen M., Yang P., Feng D., Wen T., Hu X., Pan X., Wu C. (2020). Dissolving Microneedles with Spatiotemporally Controlled Pulsatile Release Nanosystem for Synergistic Chemo-Photothermal Therapy of Melanoma. Theranostics.

[52] Anil Kumar Jeeja P., Nafisa A., Nguyen A., Eliasof A., Berger A., Loman-Cortes P., Vivero-Escoto J.L. (2025). Enhanced Photodynamic Therapy of Cancer Using Porphyrin-Based Nanoparticles Synthesized via the Co-Precipitation Method. RSC Adv..

[53] Algorri J.F., Ochoa M., Roldán-Varona P., Rodríguez-Cobo L., López-Higuera J.M. (2021). Photodynamic Therapy: A Compendium of Latest Reviews. Cancers.

[54] Wiehe A., Senge M.O. (2023). The Photosensitizer Temoporfin (mTHPC)—Chemical, Pre-Clinical and Clinical Developments in the Last Decade. Photochem. Photobiol..

[55] Nadkernichnaya E.V., Ermoschenkova M.V., Semin V.E., Parts S.A., Galikin V.N., Reshetov I.V. (2025). Photodynamic therapy of basal cell carcinoma of the face H-zone. Biomed. Photonics.

[56] Tzerkovsky D.A., Mazurenko A.N., Petrovskaya N.A., Artemyeva T.P. (2017). Photodynamic therapy with photosensitizer photolon for basal cell carcinoma. Biomed. Photonics.

[57] Krylova L.V., Otvagin V.F., Gribova G.P., Kuzmina N.S., Fedotova E.A., Zelepukin I.V., Nyuchev A.V., Kustov A.V., Morshnev P.K., Berezin D.B. (2025). Developing Chlorin/Arylaminoquinazoline Conjugates with Nanomolar Activity for Targeted Photodynamic Therapy: Design, Synthesis, SAR, and Biological Evaluation. J. Med. Chem..

[58] Reshetnickov A.V. (2014). AbeBooks. The Medicine of Light (Color): Harnessing the Healing Power of Light-Based Therapies to Overcome Cancer, Pre-Cancer, and Other Chronic Diseases.

[59] Huang J., Cai Y., Wang H. (2026). Successful Photodynamic Therapy for a Case of Cutaneous Squamous Cell Carcinoma with Difficulty in Primary Healing After Surgery. Clin. Case Rep..

[60] Maytin E.V., Kaw U., Ilyas M., Mack J.A., Hu B. (2018). Blue Light versus Red Light for Photodynamic Therapy of Basal Cell Carcinoma in Patients with Gorlin Syndrome: A Bilaterally Controlled Comparison Study. Photodiagnosis Photodyn. Ther..

[61] Galvão L.E.G., Gonçalves H.d.S., Botelho K.P., Caldas J.C. (2017). Daylight Photodynamic Therapy—Experience and Safety in Treatment of Actinic Keratoses of the Face and Scalp in Low Latitude and High Brightness Region. An. Bras. Dermatol..

[62] Patel V.A., Arron S.T., Berman B., Chapman M.S., Jambusaria-Pahlajani A., Martin G., Rossi A.M., Schlesinger T., Zeitouni N.C., Bhatia N. (2025). Expert Consensus-Based Recommendations on the Use of Photodynamic Therapy in Actinic Keratosis Patients. JAAD Int..

[63] Kaw U., Ilyas M., Bullock T., Rittwage L., Riha M., Vidimos A., Hu B., Warren C.B., Maytin E.V. (2020). A Regimen to Minimize Pain during Blue Light Photodynamic Therapy of Actinic Keratoses: Bilaterally Controlled, Randomized Trial of Simultaneous versus Conventional Illumination. J. Am. Acad. Dermatol..

[64] Godinho Ramos S., Nevitte L., Hitchens E., Stevenson P., Vilenchik V., Singh Thandi C., Keith D. (2026). Retrospective Chart Review and Clinical Experience of Using Fully Ablative Carbon Dioxide Laser Assisted Photodynamic Therapy to Treat Basal Cell Carcinoma with 2 Year Outcomes. Lasers Med. Sci..

[65] Salva K.A., Wood G.S. (2015). Epigenetically Enhanced Photodynamic Therapy (ePDT) Is Superior to Conventional Photodynamic Therapy for Inducing Apoptosis in Cutaneous T-Cell Lymphoma. Photochem. Photobiol..

